# Prior Local or Systemic Treatment: A Predictive Model Could Guide Clinical Decision-Making for Locoregional Recurrent Breast Cancer

**DOI:** 10.3389/fonc.2021.791995

**Published:** 2022-02-07

**Authors:** Huai-liang Wu, Yu-jie Lu, Jian-wei Li, Si-yu Wu, Xiao-song Chen, Guang-yu Liu

**Affiliations:** ^1^ Department of Breast Surgery, Key Laboratory of Breast Cancer in Shanghai, Shanghai Cancer Center, Fudan University, Shanghai, China; ^2^ Department of Oncology, Shanghai Medical College, Fudan University, Shanghai, China; ^3^ Department of General Surgery, Comprehensive Breast Health Center, Ruijin Hospital Shanghai Jiao Tong University School of Medicine, Shanghai, China

**Keywords:** locoregional recurrence, breast cancer, nomogram, local treatment, systemic treatment

## Abstract

**Introduction:**

Locoregional recurrent breast cancer indicates poor prognosis. No solid prediction model is available to predict prognosis and guide clinical management. Prior local treatment or systemic treatment remains controversial.

**Methods:**

Locoregional recurrent breast cancer patients operated in Fudan University Shanghai Cancer Center were enrolled as a training cohort. An external validation cohort included breast cancer patients after locoregional recurrence from Ruijin Hospital, Shanghai Jiaotong University. A nomogram predicting overall survival after locoregional recurrence was established using multivariable Cox regression analysis while internal and external validation were performed to evaluate its calibration and discrimination.

**Results:**

Overall, 346 and 96 breast cancer patients were included in the training cohort and the validation cohort separately. A nomogram was developed, including age, neoadjuvant chemotherapy, breast surgery, pathology type, tumor size, lymph node status, hormonal receptor and Her-2 status, disease-free interval, and sites of locoregional recurrence. It had modest calibration and discrimination in the training cohort, internal validation and external validation (concordance index: 0.751, 0.734 and 0.722, respectively). The nomogram classified 266 and 80 patients into low and high-risk subgroups with distinctive prognosis. Local treatment after locoregional recurrence was associated with improved overall survival in low-risk group (*P* = 0.011), while systemic therapies correlated with better outcomes only in high-risk group (*P* < 0.001).

**Conclusion:**

A nomogram based on clinicopathological factors can predict prognosis and identify low and high-risk patients. Local treatment is a prior choice for low-risk patients whereas systemic treatment needs to be considered for high-risk patients, warranting further validation and exploration.

## Introduction

Breast cancer, the most common malignant tumor in women, was estimated to have 18.1 million newly diagnosed cases and to cause 9.6 million deaths in 2018 (1). Despite the development and regulation of standard comprehensive treatment, incidence rates of locoregional recurrent breast cancer after initial operation and systemic treatments remain 7%-15% (2–5). Locoregional recurrence (LRR) from early breast cancer after mastectomy or breast-conserving treatment (BCT) plus radiotherapy indicated poor prognosis, whereas locoregional recurrent breast cancers were more likely to precede local progression and/or distant metastasis (6, 7). Many previous studies have investigated predictive factors for the LRR from early breast cancer (8–11). According to a previous review on the multidisciplinary management of LRR from breast cancer, it summarized prognostic factors of LRR from breast cancer into three parts, including patient factors (age and family history), disease features [disease-free interval (DFI), biological features, initial disease stage, and sites of LRR], and previous treatment (initial surgery, systemic treatment, radiotherapy and resectable surgery after LRR) (12).

Once LRR occurs in patients with breast cancer, whether to perform chemotherapy and the priority between local treatment and systemic therapies remain unclear and controversial (13, 14). There are few prospective clinical cohorts of local treatment or systemic treatments after LRR to guide clinicians in making preferable decisions. Unavoidable case-by-case bias in the treatment choice and efficacy estimation due to the heterogeneity of recurrent disease and previous treatment is a major obstacle for starting prospective trials on post-LRR management. To date, clinicians have usually developed treatment strategies by multidisciplinary approaches for recurrent diseases (12, 13, 15). However, it is nearly impossible for physicians to treat each recurrent case through a multidisciplinary approach team. Considering these conundrums, a comprehensive clinical tool such as predictive models for post-LRR management is critically needed.

Predictive models for post-LRR patients contributed to therapeutic implications and socioeconomic considerations. Specifically, patients whose prognosis is poor may be considered for aggressive treatments, while those with an expected long-term survival might be saved from overtreatment and its related financial burden (16, 17). However, to the best of our knowledge, no previous study has included comprehensively significant prognostic factors to develop and externally validate predictive models for post-LRR breast cancer patients. Therefore, this study aims to derive and validate a predictive model using significant clinicopathological factors to guide clinical decision-making.

## Materials and Methods

### Study Design and Participants

A retrospective two-cohort study was performed to investigate the prognosis of patients with breast cancer and the significance of local treatment and systemic treatment after LRR were evaluated. Patients with locoregional recurrent breast cancer treated between December 2007 and August 2020 in Fudan University Shanghai Cancer Center (FUSCC), Shanghai, China, were retrospectively included as a training cohort. An internal validation cohort was created by 500 bootstrap resamples of the training cohort. In addition, 96 patients with recurrent breast cancer were enrolled from the Comprehensive Breast Health Center, Ruijin Hospital, Shanghai Jiaotong University School of Medicine (RJCBHC), between January 2009 and December 2018 as an external validation cohort. The inclusion criteria were as follows: 1) patients with primary or recurrent breast cancer who were admitted to FUSCC; 2) the presence of pathologically confirmed breast cancer; 3) locoregional recurrence of breast cancer; and 4) completed breast operation (mastectomy or BCT). The exclusion criteria were described in the **Supplementary Figure S1** and included: 1) phylodes tumors; 2) without completed clinical or pathological data; 3) with distant metastasis before LRR or with the first LRR; 4) male breast cancer; 5) highly suspected second primary lesions.

### Baseline Characteristics, Follow-Up and Outcome

Patients’ characteristics in the training cohort and external validation cohort, including age at diagnosis of breast cancer (≤ 35, 35-70 or ≥ 70 years old), body mass index, menopausal status, neoadjuvant chemotherapy (NACT) received or not, initial breast operation (mastectomy or BCT), initial axillary operation (axillary lymph node dissection or sentinel lymph node biopsy), histology grade (I, II, III), pathology [ductal carcinoma *in situ*, invasive ductal carcinoma (IDC), invasive lobular carcinoma and other types], tumor size (≤ 2.0 cm or > 2.0 cm), numbers of metastatic lymph nodes (LNs) after initial operation (0, 1-3, 4-10, or >10), estrogen receptor (ER) status, progesterone receptor (PR) status, hormonal receptor (HR) status and human epidermal growth factor receptor-2 (Her-2) status, DFI (≤ 2 years or > 2 years), and sites of LRR (chest wall, breast, nodal recurrence, and multiple sites), are displayed in **Table 1**. Treatment therapies after initial operation and after LRR were showed in the **Supplementary Table S1**.

**Table 1:** Baseline characteristics of breast cancer patients with LRR.

Variable	Training cohort (%) *N* = 346	External validation cohort (%) *N* = 96	*P* value
Age at the diagnosis of breast cancer, year			0.671
≤35	35 (10.1%)	11 (11.5%)	
35-70	277 (80.1%)	73 (76.0%)	
≥70	34 (9.8%)	12 (12.5%)	
BMI			0.838
≤25	263 (76.0%)	72 (75.0%)	
>25	83 (24.0%)	24 (25.0%)	
Menopausal status			0.400
Premenopausal	142 (41.0%)	44 (45.8%)	
Postmenopausal	204 (59.0%)	52 (54.2%)	
Received NACT before surgery			0.310
No	272 (78.6%)	80 (83.3%)	
Yes	74 (21.4%)	16 (16.7%)	
Initial breast operation			0.093
BCT	89 (25.7%)	33 (34.4%)	
Mastectomy	257 (74.3%)	63 (65.6%)	
Initial axillary operation			0.115
Only SLNB	100 (28.9%)	38 (39.6%)	
ALND ± SLNB	238 (68.8%)	55 (57.3%)	
No axillary operation	8 (2.3%)	3 (3.1%)	
Histology grade			0.643
I	1 (0.3%)	1 (1.0%)	
II	100 (28.9%)	29 (30.2%)	
III	168 (48.6%)	49 (51.0%)	
Unknown	77 (22.3%)	17 (17.7%)	
Pathology			<0.001^***^
IDC	258 (74.6%)	87 (90.6%)	
ILC	5 (1.4%)	4 (4.2%)	
Others	83 (24.0%)	5 (5.2%)	
Tumor size, cm			0.567
≤2.0	170 (49.1%)	44 (45.8%)	
>2.0	176 (50.9%)	52 (54.2%)	
Positive LN			0.317
0	164 (47.4%)	46 (47.9%)	
1-3	82 (23.7%)	24 (25.0%)	
4-9	59 (17.1%)	10 (10.4%)	
≥10	41 (11.8%)	16 (16.7%)	
ER status			0.920
Negative	171 (49.4%)	48 (50.0%)	
Positive	175 (50.6%)	48 (50.0%)	
PR status			0.011^*^
Negative	195 (56.4%)	68 (70.8%)	
Positive	151 (43.6%)	28 (29.2%)	
HR status			0.726
Negative	166 (48.0%)	48 (50.0%)	
Positive	180 (52.0%)	48 (50.0%)	
HER-2 status			0.979
Negative	241 (69.7%)	67 (69.8%)	
Positive	105 (30.3%)	29 (30.2%)	
DFI to LRR			0.999
≤2 year	191 (55.2%)	53 (55.2%)	
>2 year	155 (44.8%)	43 (44.8%)	
Sites of LRR			0.441
Chest wall	129 (37.3%)	25 (26.0%)	
Breast	64 (18.5%)	33 (34.4%)	
Nodal recurrence	123 (35.5%)	31 (32.3%)	
Multiple sites	30 (8.7%)	7 (7.3%)	

*indicates P < 0.05; ***indicates P < 0.001.

ALND, Axillary lymph node dissection; BCT, Breast-conserving treatment;

BMI, Body mass index; DCIS, Ductal carcinoma in situ; DFI, Disease-free Interval;

ER, Estrogen receptor; Her-2, Human epidermal growth factor receptor-2;

HR, Hormonal receptor; IDC, Invasive ductal carcinoma; ILC, Invasive lobular carcinoma;

LN, Lymph node; LRR, Locoregional recurrence; NACT, Neoadjuvant chemotherapy treatment;

PR, Progesterone receptor; SLNB, Sentinel lymph node biopsy.

ER and PR positivity were defined according to our previous studies (18). HR positivity was defined as the positivity of either ER or PR. The DFI was calculated from the time interval from the initial operation to the occurrence of the first LRR. LRR referred to breast cancer recurrence in the ipsilateral chest wall or breast or regional lymph nodes (axillary lymph node, clavicular lymph node and internal mammary lymph node) after excluding highly suspected second primary lesions. Besides, highly suspected second primary lesions were defined as that the recurrent tumors were found in the different quadrant or far from the primary tumor scar in isolated ipsilateral local recurrent patients (breast and chest wall), and patients with inconsistent immunohistochemistry status in reginal nodal recurrent patients (12). Multiple sites indicated that recurrent sites occurred in more than one region mentioned above. Distant metastasis (DM) referred to tumor recurrence outside the locoregional areas mentioned above. Patients were censored when DM or death occurred or were lost to follow-up. Distant disease-free survival (DDFS) and overall survival (OS) after LRR were calculated from the time of the first LRR.

### Statistical Analysis

Comparison of clinicopathological characteristics was evaluated using the χ^2^ test or Fisher’s exact test for categorical variables between the training cohort and validation cohort. DDFS and OS were evaluated using the Kaplan-Meier method, and the differences were compared using the unstratified log-rank test. Univariate and multivariate Cox regression were used to screen the risk factors. LASSO Cox regression analysis was implemented to further confirm the candidate prognostic factors and complete the construction of the predictive model.

**Figure 1:**
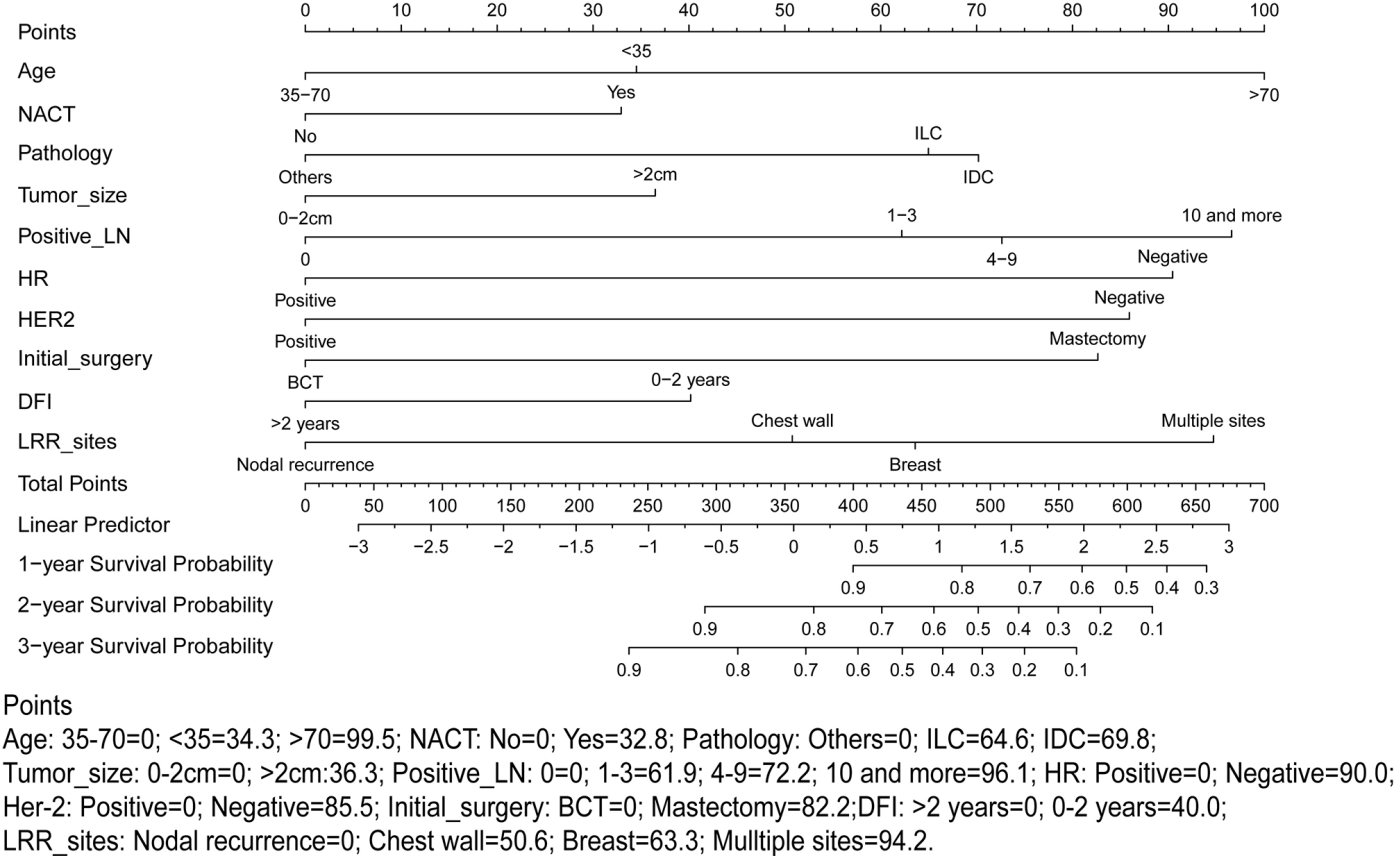
Proposed nomogram using the training cohort to predict the probability of overall survival (OS) after locoregional recurrence (LRR). NACT, Neoadjuvant chemotherapy treatment; LN, Lymph node; HR, Hormonal receptor; HER-2, human epidermal growth factor receptor-2; LRR, Locoregional recurrence; DFI, Time interval from initial surgery to the LRR.

A nomogram predicting OS was formulated based on the results of multivariate analysis and expert consensus. Model performance was assessed in the training cohort, internal validation cohort and external validation cohort through discrimination and calibration. Discrimination ability was assessed by receiver operating characteristic (ROC) analysis, and predictive accuracy was measured using the concordance index (C-index). The C-index ranges from 0.5 to 1.0 (random to perfect prediction) (19). Calibration analysis was performed through the comparison between predicted probabilities and actual probabilities. For predictive factors included in the nomogram, each value represented a score on the point axis. A total score is calculated by adding the scores for each item and locating this sum on the total point scale axis. The three vertical lines can be used to predict the probability of OS within 1 year, 2 years and 3 years (**Figure 1**). We used X-tile, a type of bioinformatics tool, to determine the appropriate cutoff points of predictive scores to stratify patients into low-risk and high-risk subgroups (20). All hypothesis tests were two-sided, and *P* < 0.05 was considered statistically significant. Statistical analyses were performed using SPSS statistical software (v22.0) and R statistical software (v3.5.2). The R packages used in this study are as follows: ‘survival’, survminer’, ‘rms’, ‘riskRegression’, ‘maxstat’, ‘pROC’, ‘plotROC’, ‘ggplot2’, and ‘nomogramFormula’.

## Results

### Baseline Characteristics

A total of 346 patients in the training cohort and 96 patients in the external validation cohort who underwent mastectomy or BCT were included in the final analysis. The median follow-up after the initial operation was 57.1 months (range 5.3-152.3 months) in the FUSCC cohort and 49.8 months (range 12.77-113.57 months) in the RJCBHC cohort. Clinicopathological characteristics were similar between the training cohort and external validation cohort except for pathology and PR status (**Table 1**). Most of the patients had IDC in the external validation cohort, while the proportion of IDC in the training cohort was obviously lower (90.6% *vs* 74.6%, *P* < 0.001). In addition, therapeutic choices seemed to be significantly different between these two cohorts (**Supplementary Table S1**). The median follow-up period after LRR was 29.1 months (range 0.1-134.4 months), while it was 25.1 months (range 3.4-85.8 months) in the external validation cohort. There were obviously higher death and DM rates in the training cohort (death rate: 33.5% *vs* 14.6%; DM rate 30.1% *vs* 6.3%).

### Identified Prognostic Factors for Post-LRR Outcomes

The results of the univariate analysis and potential high-risk factors are listed in **Supplementary Table S2**. Multivariate analyses demonstrated that age group, NACT, pathology type, larger tumor size, metastatic lymph nodes, HR status, and Her-2 status were significantly associated with poor prognosis for post-LRR patients (**Table 2**).

**Table 2:** Multivariate cox regression analysis of prognostic factors in the training cohort.

Variable	Distant-disease free survival (DDFS)			Overall survival (OS)		
	Hazard Ratio	95% Confidence Interval	*P* value	Hazard Ratio	95% Confidence Interval	*P* value
Age of diagnosis of breast cancer, yrs						
35-70	1.000 (reference)	—	—	1.000 (reference)	—	—
≤35	1.358	0.834-2.210	0.218	1.234	0.685-2.223	0.484
≥70	1.830	1.066-3.144	0.029^*^	2.219	1.205-4.087	0.011*
Receive NACT						
No	1.000 (reference)	—	—	1.000 (reference)	—	—
Yes	1.793	1.216-2.643	0.003^**^	1.519	0.950-2.430	0.081
Initial breast operation						
BCT	1.000 (reference)	—	—	1.000 (reference)	—	—
Mastectomy	1.096	0.572-2.100	0.783	2.102	0.756-5.844	0.154
Pathology						
IDC	1.000 (reference)	—	—	1.000 (reference)	—	—
ILC	1.014	0.243-4.237	0.984	1.000	0.134-7.443	1.000
Others	0.562	0.372-0.851	0.006^**^	0.485	0.289-0.813	0.006**
Tumor size						
≤2.0	1.000 (reference)	—	—	1.000 (reference)	—	—
>2.0	1.479	1.042-2.101	0.029^*^	1.438	0.945-2.187	0.090
Number of positive LNs						
0	1.000 (reference)	—	—	1.000 (reference)	—	—
1-3	1.648	1.088-2.494	0.018^*^	1.924	1.155-3.207	0.012*
4-9	2.058	1.276-3.320	0.003^**^	2.189	1.232-3.890	0.008^**^
≥10	2.527	1.543-4.141	<0.001^***^	2.601	1.453-4.656	0.001^**^
HR status						
Negative	1.000 (reference)	—	—	1.000 (reference)	—	—
Positive	0.540	0.324-0.899	0.018^*^	0.273	0.142-0.523	<0.001^***^
Her-2 status						
Negative	1.000 (reference)	—	—	1.000 (reference)	—	—
Positive	0.495	0.341-0.720	<0.001^***^	0.415	0.263-0.653	<0.001^**^
Adjuvant radiotherapy						
No	1.000 (reference)	—	—	1.000 (reference)	—	—
Yes	0.686	0.480-0.981	0.039^*^	0.676	0.444-1.029	0.068
Hormonal therapy						
No	1.000 (reference)	—	—	1.000 (reference)	—	—
Yes	1.597	0.955-2.672	0.074	1.678	0.889-3.169	0.110
DFI to LRR						
≤2, yrs	1.000 (reference)	—	—	1.000 (reference)	—	—
>2, yrs	0.770	0.546-1.087	0.138	0.662	0.443-1.012	0.057
Locoregional recurrence sites						
Breast	1.000 (reference)	—	—	1.000 (reference)	—	—
Chest wall	1.230	0.553-2.737	0.611	0.894	0.280-2.850	0.849
Nodal recurrence	1.188	0.567-2.490	0.648	0.563	0.184-1.720	0.313
Multiple sites	1.689	0.715-3.993	0.232	1.280	0.372-4.403	0.696

*indicates P < 0.05; **indicates P < 0.01; ***indicates P < 0.001.

NACT, Neoadjuvant chemotherapy treatment; BCT, Breast-conserving treatment; IDC, Invasive ductal carcinoma; ILC, Invasive lobular carcinoma; LN, Lymph node; HR, Hormonal receptor; Her-2, human epidermal growth factor receptor-2; DFI, Disease-free Interval; LRR, Locoregional recurrence.

### Performance of the Predictive Nomogram

According to the high-risk factors identified in previous studies (12, 21), a nomogram predicting the probability of OS based on multivariate Cox regression analysis and expert opinions was constructed (**Figure 1**). A total score was calculated using age at the diagnosis of breast cancer, received NACT or not, pathology types, tumor size, number of positive lymph nodes, HR status, HER-2 status, type of initial breast operation, DFI, and location of LRR. Discrimination assessment showed good performance of the model (C-index: 0.751 and AUC: 0.775 [0.705-0.845]) and stable agreement (C-index: 0.734 and AUC: 0.774 [0.710-0.838]) in the original training and internal validation cohorts, respectively. However, this nomogram seemed to underestimate the OS probability (C-index: 0.722 and AUC: 0.679 [0.536-0.823]) in the external validation cohort (**Supplementary Figure S2**).

### Clinical Implications of the Predictive Nomogram

**Figure 2:**
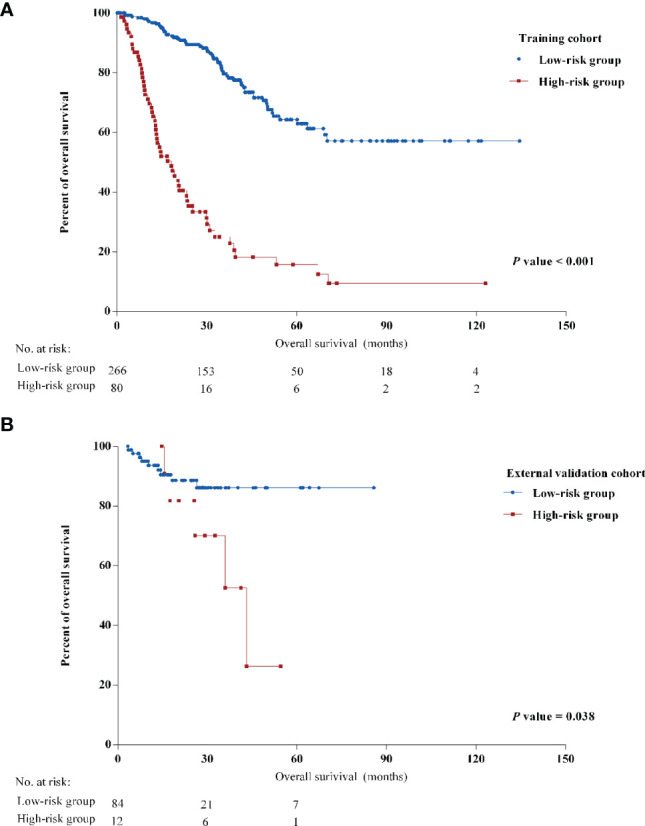
Kaplan-Meier curves of overall survival (OS) of different risk groups in **(A)** the training cohort and **(B)** the external validation cohort.

Stratified by the nomogram model, we divided post-LRR patients into a low-risk group and a high-risk group (the cutoff point was 441.4). Stratification into low-risk and high-risk subgroups allowed significant distinction between the Kaplan-Meier curves for survival outcomes in both the training cohort and the external validation cohort (**Figure 2**). Clinicopathological characteristics were significantly different between the low-risk group and the high-risk group in the training cohort (**Supplementary Table S3**). Most high-risk patients presented with negative HR and Her-2 status (80.0% and 83.8%, respectively), while more than half (61.7%) were luminal breast cancer patients in the low-risk group.

**Figure 3:**
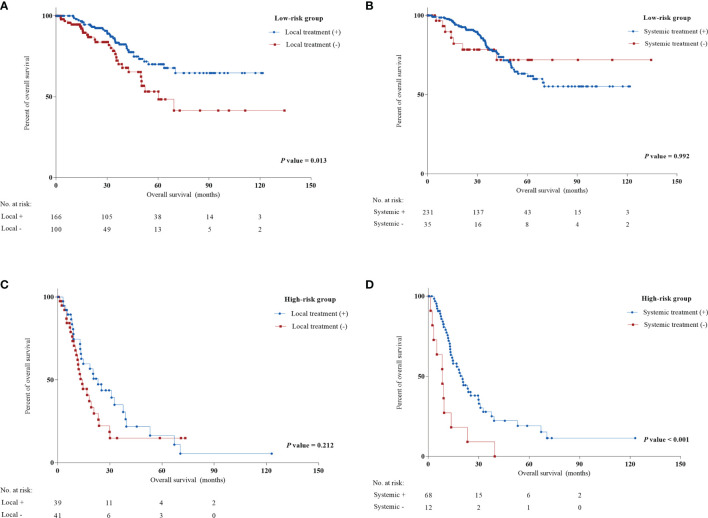
Univariate Cox regression analysis of local treatment and systemic treatment in low and high-risk groups from the training cohort. **(A)** Local treatment in low-risk group; **(B)** systemic treatment in low-risk group; **(C)** local treatment in high-risk group; **(D)** systemic treatment in high-risk group.

To evaluate the priority of different treatment therapies after LRR, we examined the association of different therapies and prognosis in low-risk and high-risk patients. **Figure 3** indicates the significance of local treatment in the low-risk group and systemic treatment in the high-risk group (HR: 0.513 [0.303-0.856] and 0.182 [0.085-0.387]). Specifically, resectable surgery seemed to be associated with longer survival for low-risk patients (HR: 0.548, *P* = 0.029) (**Supplementary Table S4**). Interestingly, chemotherapy and hormonal therapy in the high-risk group improved OS (HR: 0.386 and 0.200, *P* = 0.011 and 0.001, respectively). In contrast, chemotherapy seemed to not be associated with improved OS in the low-risk group, while resectable surgery did not correlate with better prognosis for high-risk patients. No significant benefits in OS were observed for radiotherapy in either the low-risk or high-risk group. However, radiotherapy following LRR was correlated with increased DDFS in the low-risk group but not in the high-risk group (*P* = 0.024 and 0.623, respectively) (**Supplementary Figure S3**).

**Figure 4:**
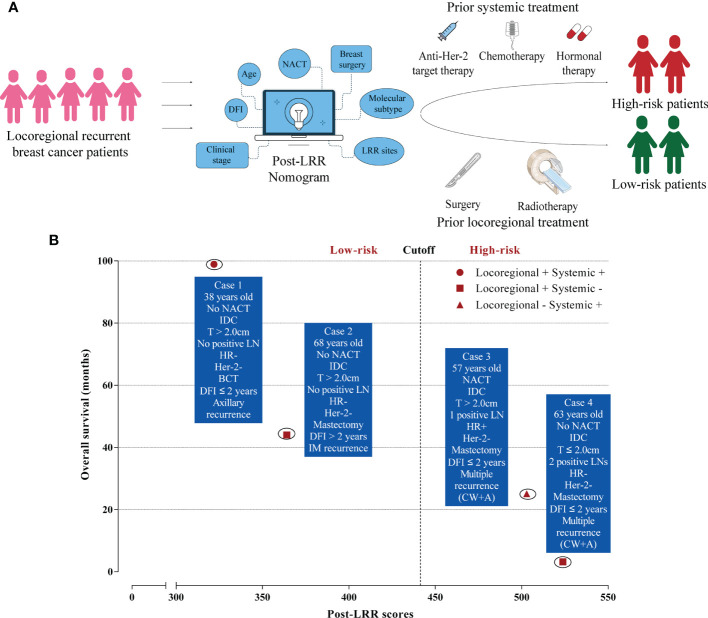
Clinical management of locoregional recurrent breast cancer patients. **(A)** The schematic diagram of post-locoregional recurrent breast cancer patient management based on the nomogram; **(B)** Examples of low and high-risk patients in real-world practice. LRR, Locoregional recurrence; DFI, Disease-free interval; NACT, Neoadjuvant chemotherapy; IDC, Invasive ductal carcinoma; LN, Lymph node; HR, Hormonal receptor; BCT, Breast-conserving treatment; IM, Internal mammary; CW, Chest wall; A, Axillary.

To conclude that a schematic diagram of clinical management of post-LRR breast cancer patients based on the nomogram is illustrated (**Figure 4A**). Once locoregional recurrent breast cancer patients come to clinic, the nomogram could stratify these patients into low-risk or high-risk groups and provide prior treatment strategies for them. Furthermore, it is displayed that there are several examples of low-risk or high-risk patients in real-world practice (**Figure 4B**). For these two high-risk cases, multiple recurrent sites, negativity of Her-2, and short DFI were observed. However, it is inconsistent with our previous perception, post-LRR luminal breast cancer patient (Case 3) with only one positive lymph node is regarded as a high-risk patient. Case 3 was still alive after receiving chemotherapy and hormonal therapy while case 4 only receiving radiotherapy was died within 4 months after LRR. This indicated the huge significance of systemic therapies for high-risk patients.

## Discussion

Our study demonstrated the relative importance of a breadth of high-risk prognostic variables for locoregional recurrent breast cancer patients, such as age at diagnosis of breast cancer, NACT, pathology type, tumor size, number of metastatic LNs, HR status, and Her-2 status. Furthermore, we constructed a nomogram to target and stratify patients into low-risk and high-risk subgroups. Local treatment was found to be correlated with improved survival in the low-risk group but not in the high-risk group. In contrast, systemic treatment is associated significantly improved OS only for high-risk patients. We expect that this predictive nomogram will prove helpful in further clinical practice on post-LRR management.

The nomogram reported and validated here was constructed to quantify the risks of several clinical profiles mentioned above. Through the nomogram, we found that the occurrence of LRR in multiple sites, 10 or more metastatic lymph nodes and age greater than 70 years were prognostic factors for poor OS. Similar findings were observed in previous studies (21–23). Interestingly, mastectomy was found to be a high-risk factor for post-LRR outcomes in the nomogram. The Danish 82b/c trials and another postmastectomy trial in British Columbia also found that the development of LRR and receipt of initial mastectomy were likely to precede metastatic disease (24, 25). Hence, prognosis of patients with LRR was significantly correlated with therapeutic choices before the occurrence of LRR. A consistent finding was noted in a recent clinical trial that intraoperative radiotherapy before LRR could achieve better prognosis compared to those receiving whole-breast external beam radiotherapy before (26). A previous risk stratification system using three robust risk factors, including positive LN status *vs* negative, DFI < 30 months *vs* ≥ 30 months and regional LN recurrence *vs* local recurrence, could guide patients in making a choice with estimated survival (21). However, in our nomogram, nodal recurrence seemed to be a protective factor for post-LRR patients. A possible explanation for this was the heterogeneity of different nodal recurrences. The survival data of the training cohort indicated that the OS of internal mammary LN and axillary LN recurrence was significantly better than that of chest wall recurrence, while clavicular LN recurrence showed an obviously adverse prognosis (**Supplementary Figure S4**). Furthermore, axillary recurrence was observed as a worse prognostic factor compared to those isolated breast recurrences. Jin et al. indicated a consistent finding that the 5-year OS of isolated breast recurrence was significantly higher than that of axillary nodal recurrence (100% *vs* 73.5%, p=0.021) (27).

Using another similar risk stratification system, Byoung Hyuck Kim et al. screened and targeted post-LRR patients with long-term survival through the initial pN stage, DFI interval and whether to perform resectable excision after LRR (15). Our study constructed a nomogram using more prognostic factors with internal and external validation to quantify the impacts of these prognostic factors in predicting OS. Moreover, it could estimate the probability of OS for patients and help them achieve a balance between potential benefits from treatments and socioeconomic factors such as financial cost and family considerations (28, 29). After further analysis on two different types of isolated local recurrence separately, similar findings were observed compared to the results reported above, which might demonstrate the stability of this predictive model (**Supplementary Table S5**).

The latest ESO-ESMO consensus for advanced breast cancer supported surgical excision if feasible for patients and if the recurrent sites are resectable (13). No surprisingly, resectable surgery was found to be associated with better OS in the low-risk group. However, some local recurrences involve extensive chest wall recurrence, and some regional recurrences occurring in surgically inaccessible sites cannot receive resectable surgery in the clinic. Anders N. Pedersen et al. found that the complete remission rate in resectable surgery was obviously higher than that in patients not given surgery (76% *vs* 43%, *P* < 0.0001) (30). In contrast, previous studies suggested that favorable prognosis correlated with the availability of resectable surgery (12, 31). Therefore, it is still unclear whether resectable surgery or resectable LRR originally contributed to a better prognosis. Radiotherapy was indicated for patients not previously irradiated, and the standard of reirradiation was still controversial (13). Through this nomogram, we could target patients with an expected poor prognosis (low-risk group). Further univariable analysis proved the significance of local treatment for low-risk patients. Although radiotherapy could not significantly improve OS, it was found to have a positive role in prolonging DDFS in the low-risk group (**Supplementary Figure S3**). Low-risk patients seemed to not obtain significant benefits from chemotherapy and other systemic treatments. Our findings were not only consistent with previous studies (26, 32) but also provided new therapeutic approaches for post-LRR patients.

In contrast, chemotherapy was only found to be correlated with longer survival for high-risk patients in our study. This finding was consistent with the risk stratification study mentioned above (15). Previously, the CALOR trial confirmed the positive role of chemotherapy in ER-negative patients but not in ER-positive patients (33). Owing to the limitations of the CALOR trial, it should not be concluded that all ER-positive patients cannot gain benefits from chemotherapy (33). Therefore, this study provides a new strategy to determine whether to use chemotherapy. Furthermore, except for chemotherapy, other systemic treatments should be considered based on their previous medical history and patient status (12, 13, 32). Tamoxifen improved the 5-year DFS of ER-positive LRR patients in the SAKK 23/82 trial (34). To date, no prospective trial has investigated the role of anti-Her-2 targeted therapy in Her-2-positive LRR patients. However, most experts still recommend the use of anti-Her-2 targeted therapy for post-LRR patients with Her-2 positivity as a standard treatment. Thus, with the combination of our findings and previous studies as well as expert consensus, physicians need to perform systemic therapies once patients meet the high-risk status in the clinic. This study also offers several real-world cases of both low and high-risk patients with therapeutic choices and survival after LRR to further support the clinical strategies mentioned above (**Figure 4B**).

To the best of our knowledge, this is the first study to establish a nomogram using many important prognostic factors from a relatively large training cohort accompanied by external validation to estimate the prognosis of post-LRR breast cancer patients. This nomogram showed modest performance and generalization in both internal and external validation. Moreover, based on the estimation of survival probability, patients could be divided into two different groups and had corresponding therapeutic strategies. Patients could clearly consider and decide their own treatment therapies according to the expected survival reported by the nomogram and their willingness.

Despite these important advantages, this study had several limitations. First, as a retrospective study, it would unavoidably have selection bias. It was also unable to investigate the correlation between clinicopathological factors and occurrence of LRR after initial surgery without the clinical data of all patients treated in the same period. Second, therapeutic choices and incidence rates of events were obviously different between the training and external validation cohorts, causing worse agreement in the external validation. Third, owing to the technical limitation, it’s inevitable to exclude all second primary breast tumor patients apart in our study. Finally, our nomogram dealt only with clinicopathologic profiles and needed longer follow-up. This nomogram should be applied with caution until validated in a randomized clinical trial with different treatment strategies in the future.

## Conclusion

The post-LRR predictive nomogram was developed and externally validated for patients with breast cancer and could guide oncologists in making prognosis-related clinical decisions. Local treatments following LRR could be initial choices rather than systemic treatment for low-risk patients, while systemic treatment might be considered once identified as high-risk patients.

## Data Availability Statement

The datasets that support the findings of this study are kept in institutional file storage on an internal server at the Fudan University Shanghai Cancer Centre Department of breast cancer. In order to protect patient privacy, these data were unavailable to public. The data will be made available on reasonable request from the corresponding author. For all data requests, please contact Dr Guang-yu Liu (Department of Breast Surgery, Fudan University Shanghai Cancer Centre, Shanghai, P.R. China. liugy688@163.com) or Dr Xiao-song Chen (Comprehensive Breast Health Center, Ruijin Hospital, Shanghai Jiao Tong University School of Medicine, Shanghai, China. chenxiaosong0156@hotmail.com).

## Ethics Statement

Ethical approval was not provided for this study on human participants because as a retrospective cohort study, all the procedures performed in studies involving human participants were in accordance with the ethical standards of the institutional and/or national research committee and with the 1964 Helsinki declaration and its later amendments or comparable ethical standards. Written informed consent for participation was not required for this study in accordance with the national legislation and the institutional requirements.

## Author Contributions

Conceptualization: X-sC and G-yL. Data curation and formal analysis: H-lW and Y-jL. Investigation: H-lW, Y-jL, J-wL, and S-yW. Methodology: H-lW, Y-jL, J-wL, and S-yW. Project administration and supervision: X-sC and G-yL. Validation: H-lW and Y-jL. Visualization: H-lW and Y-jL. Writing - original draft: H-lW and Y-jL. Writing - review and editing: H-lW, Y-jL, J-wL, S-yW, X-sC, and G-yL. All authors contributed to the article and approved the submitted version.

## Conflict of Interest

The authors declare that the research was conducted in the absence of any commercial or financial relationships that could be construed as a potential conflict of interest.

## Publisher’s Note

All claims expressed in this article are solely those of the authors and do not necessarily represent those of their affiliated organizations, or those of the publisher, the editors and the reviewers. Any product that may be evaluated in this article, or claim that may be made by its manufacturer, is not guaranteed or endorsed by the publisher.
