# Supplementary figures and images for: Prior Local or Systemic Treatment: A Predictive Model Could Guide Clinical Decision-Making for Locoregional Recurrent Breast Cancer
Source: Front Oncol. 2022 Feb 7;11:791995. doi: 10.3389/fonc.2021.791995 (PMC8858965; doi:10.3389/fonc.2021.791995)

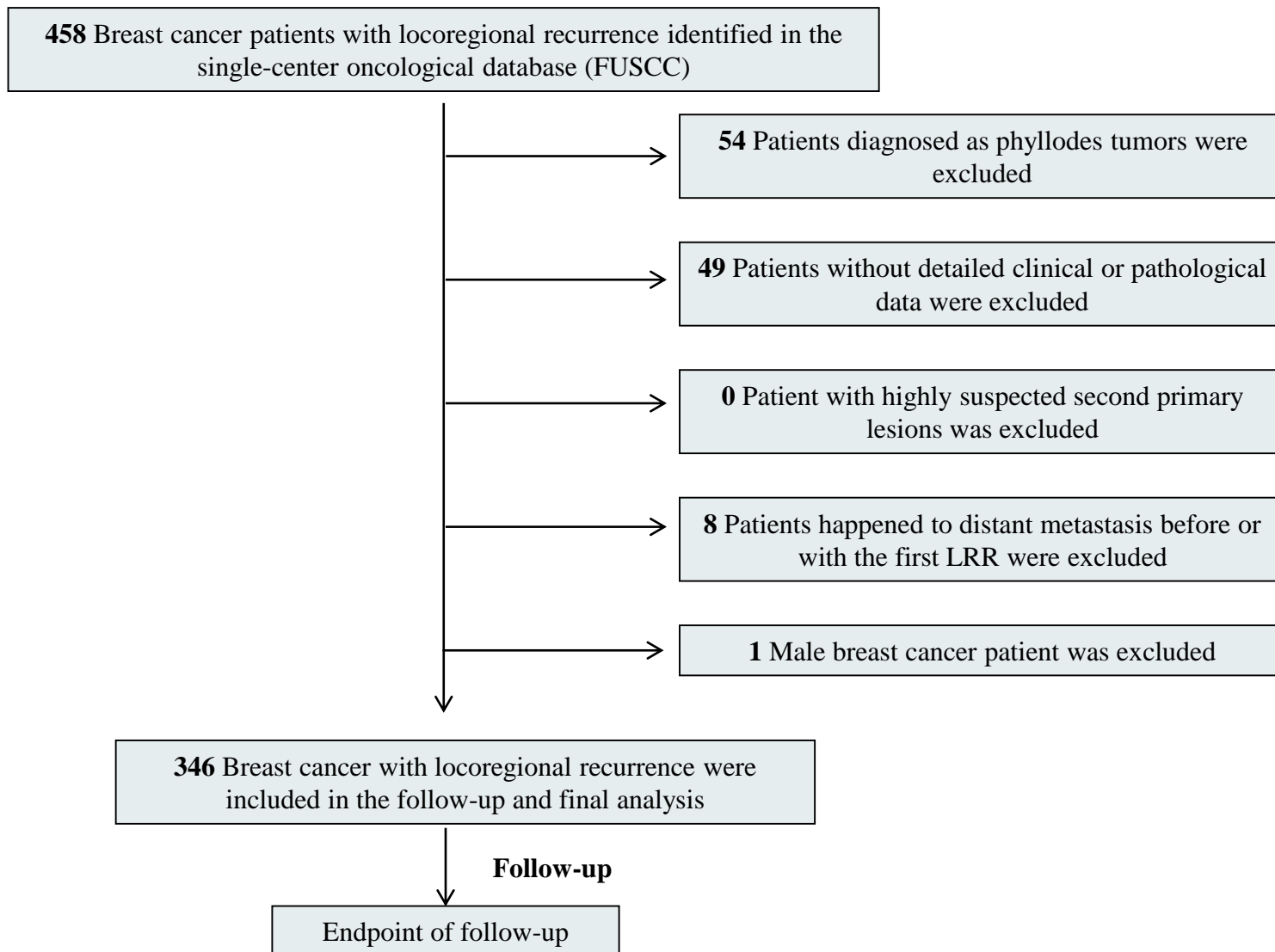

Supplement: Supplementary file 1 [file Image_1.pdf]

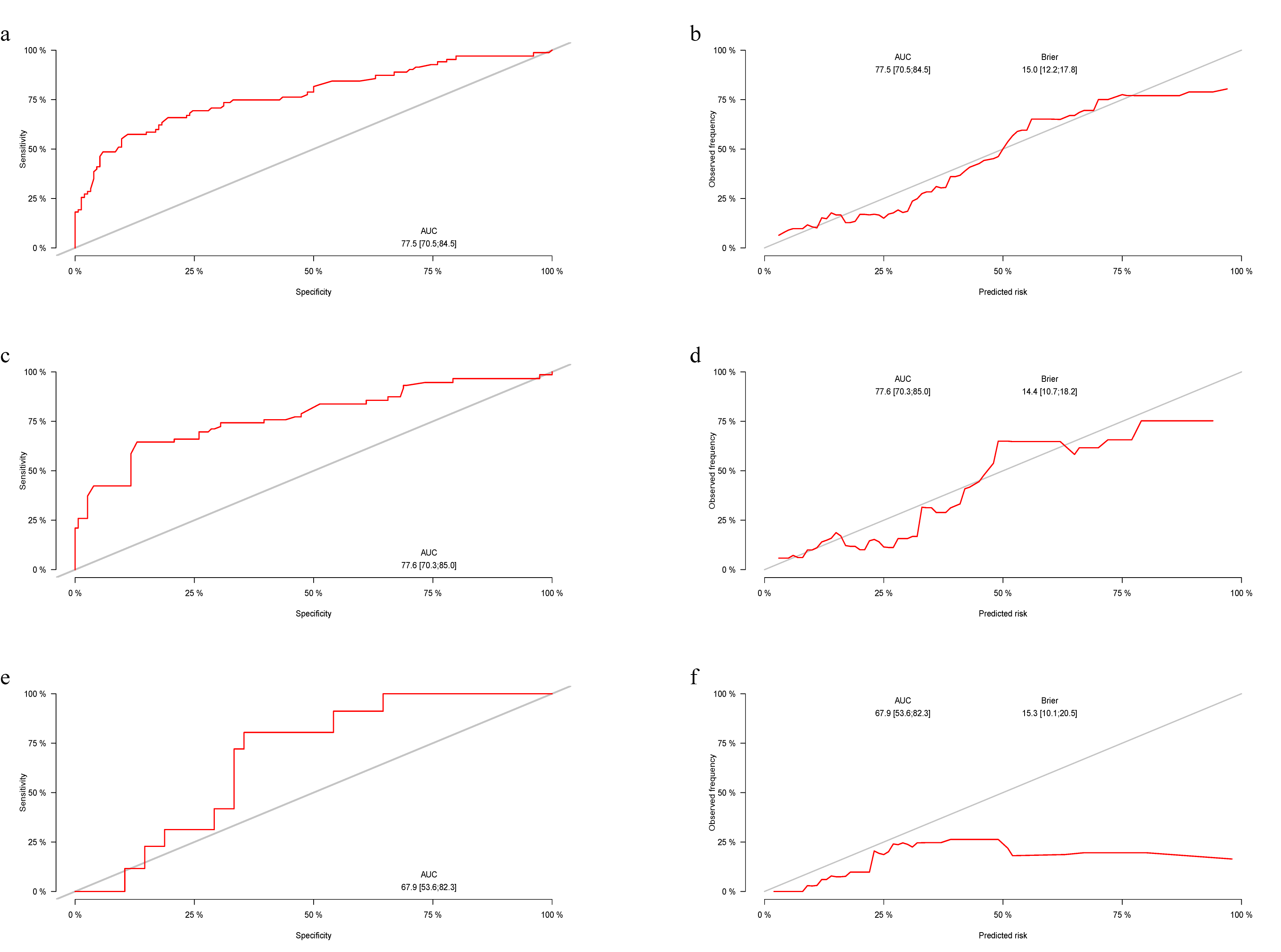

Supplement: Supplementary file 2 [file Image_2.tif]

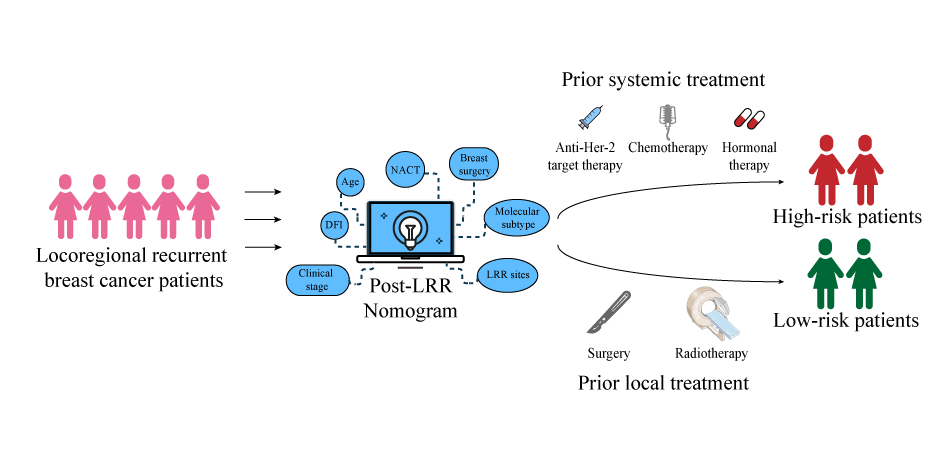

Supplement: Supplementary file 5 [file Image_5.tif]
