# Supplementary material for: Prior Local or Systemic Treatment: A Predictive Model Could Guide Clinical Decision-Making for Locoregional Recurrent Breast Cancer
Source: Front Oncol. 2022 Feb 7;11:791995. doi: 10.3389/fonc.2021.791995 (PMC8858965; doi:10.3389/fonc.2021.791995)
Supplement: Supplementary file 6 [file Table_1.docx]

**Supplementary table S1. Therapeutic choices of breast cancer patients with LRR after initial operation and LRR.**

| Variable | Training cohort (%) *N*=346 | External validation cohort (%) *N*=96 | *P* value |
| --- | --- | --- | --- |
| After initial operation |  |  |  |
| Treatment therapy |  |  |  |
| Adjuvant radiotherapy | 166 (48.0%) | 50 (52.1%) | 0.476 |
| Adjuvant chemotherapy | 269 (77.7%) | 62 (64.6%) | 0.009^**^ |
| Adjuvant hormonal therapy | 165 (47.7%) | 37 (38.5%) | 0.111 |
| Anti-Her-2 target therapy | 72 (20.8%) | 20 (20.8%) | 0.996 |
| After LRR |  |  |  |
| Treatment therapy |  |  |  |
| Local treatment |  |  |  |
| Resectable surgery | 134 (38.7%) | 31 (32.3%) | 0.249 |
| Radiotherapy | 112 (32.4%) | 20 (20.8%) | 0.029^*^ |
| Systemic treatment |  |  |  |
| Chemotherapy | 237 (68.5%) | 50 (52.1%) | 0.003^**^ |
| Hormonal therapy | 147 (42.5%) | 33 (34.4%) | 0.152 |
| Anti-Her-2 therapy | 97 (28.0%) | 15 (15.6%) | 0.013^*^ |

*indicates *P* < 0.05; **indicates *P* < 0.01; ***indicates *P* < 0.001

LRR: Locoregional recurrence.

**Supplementary table S2. Univariate cox regression analysis of the Association Between Clinicopathologic Factors, and Distant disease-free Survival (DDFS) and Overall Survival (OS) in the training cohort.**

| Variable | Distant-disease free survival (DDFS) | | |  | Overall survival (OS) | | |
| --- | --- | --- | --- | --- | --- | --- | --- |
|  | Hazard Ratio | 95% Confidence Interval | *P* value |  | Hazard Ratio | 95% Confidence Interval | *P* value |
| Age of diagnosis of breast cancer, yrs | | |  |  |  |  |  |
| 35-70 | 1.000 (reference) | — | — |  | 1.000 (reference) | — | — |
| ≤35 | 1.588 | 1.017-2.481 | 0.042^*^ |  | 1.651 | 0.967-2.818 | 0.065 |
| ≥70 | 1.722 | 1.048-2.828 | 0.032^*^ |  | 2.069 | 1.172-3.651 | 0.012^*^ |
| Menopausal status | | |  |  |  |  |  |
| Premenopausal | 1.000 (reference) | — | — |  | 1.000 (reference) | — | — |
| Postmenopausal | 1.307 | 0.951-1.795 | 0.099 |  | 1.377 | 0.939-2.020 | 0.101 |
| Receive NACT |  |  |  |  |  |  |  |
| No | 1.000 (reference) | — | — |  | 1.000 (reference) | — | — |
| Yes | 1.953 | 1.391-2.741 | <0.001^***^ |  | 1.938 | 1.292-2.909 | 0.001^**^ |
| Initial breast operation | | |  |  |  |  |  |
| BCT | 1.000 (reference) | — | — |  | 1.000 (reference) | — | — |
| Mastectomy | 1.838 | 1.237-2.732 | 0.003^**^ |  | 2.581 | 1.500-4.440 | 0.001^**^ |
| Pathology | | |  |  |  |  |  |
| IDC | 1.000 (reference) | — | — |  | 1.000 (reference) | — | — |
| ILC | 0.684 | 0.169-2.767 | 0.594 |  | 0.498 | 0.069-3.576 | 0.488 |
| Others | 0.638 | 0.431-0.943 | 0.024^*^ |  | 0.587 | 0.359-0.960 | 0.034^*^ |
| Tumor size |  |  |  |  |  |  |  |
| ≤2.0 | 1.000 (reference) | — | — |  | 1.000 (reference) | — | — |
| >2.0 | 1.731 | 1.267-2.365 | <0.001^***^ |  | 1.786 | 1.227-2.600 | 0.002^**^ |
| Number of positive LNs | | |  |  |  |  |  |
| 0 | 1.000 (reference) | — | — |  | 1.000 (reference) | — | — |
| 1-3 | 1.707 | 1.148-2.538 | 0.008^**^ |  | 1.725 | 1.062-2.801 | 0.027^*^ |
| 4-9 | 2.112 | 1.370-3.255 | 0.001^**^ |  | 2.336 | 1.392-3.920 | 0.001^**^ |
| ≥10 | 2.934 | 1.905-4.520 | <0.001^***^ |  | 3.042 | 1.836-5.039 | <0.001^***^ |
| ER status | | |  |  |  |  |  |
| Negative | 1.000 (reference) | — | — |  | 1.000 (reference) | — | — |
| Positive | 0.802 | 0.591-1.089 | 0.157 |  | 0.511 | 0.352-0.742 | <0.001*** |
| PR status | | |  |  |  |  |  |
| Negative | 1.000 (reference) | — | — |  | 1.000 (reference) | — | — |
| Positive | 0.869 | 0.637-1.185 | 0.375 |  | 0.546 | 0.371-0.803 | 0.002** |
| HR status | | |  |  |  |  |  |
| Negative | 1.000 (reference) | — | — |  | 1.000 (reference) | — | — |
| Positive | 0.774 | 0.570-1.051 | 0.100 |  | 0.505 | 0.348-0.731 | <0.001*** |
| Her-2 status | | |  |  |  |  |  |
| Negative | 1.000 (reference) | — | — |  | 1.000 (reference) | — | — |
| Positive | 0.761 | 0.541-1.069 | 0.115 |  | 0.831 | 0.576-1.198 | 0.321 |
| Adjuvant radiotherapy | | |  |  |  |  |  |
| No | 1.000 (reference) | — | — |  | 1.000 (reference) | — | — |
| Yes | 0.845 | 0.622-1.148 | 0.282 |  | 0.926 | 0.590-1.453 | 0.738 |
| Adjuvant chemotherapy | | |  |  |  |  |  |
| No | 1.000 (reference) | — | — |  | 1.000 (reference) | — | — |
| Yes | 1.056 | 0.751-1.484 | 0.755 |  | 1.130 | 0.721-1.771 | 0.595 |
| Hormonal therapy | | |  |  |  |  |  |
| No | 1.000 (reference) | — | — |  | 1.000 (reference) | — | — |
| Yes | 0.756 | 0.553-1.033 | 0.079 |  | 0.618 | 0.427-0.894 | 0.011^*^ |
| Anti-Her-2 target therapy | | |  |  |  |  |  |
| No | 1.000 (reference) | — | — |  | 1.000 (reference) | — | — |
| Yes | 0.968 | 0.693-1.352 | 0.848 |  | 0.640 | 0.387-1.059 | 0.082 |
| DFI to LRR | | |  |  |  |  |  |
| ≤2, yrs | 1.000 (reference) | — | — |  | 1.000 (reference) | — | — |
| >2, yrs | 0.710 | 0.519-0.971 | 0.032^*^ |  | 0.518 | 0.351-0.763 | 0.001^**^ |
| Locoregional recurrence sites | | |  |  |  |  |  |
| Breast | 1.000 (reference) | — | — |  | 1.000 (reference) | — | — |
| Chest wall | 2.244 | 1.367-3.685 | 0.001^**^ |  | 2.986 | 1.606-5.551 | <0.001^***^ |
| Nodal recurrence | 1.759 | 1.056-2.927 | 0.030^*^ |  | 1.283 | 0.576-2.856 | 0.542 |
| Multiple sites | 3.304 | 1.705-6.402 | <0.001^***^ |  | 4.357 | 1.918-9.899 | <0.001^***^ |

*indicates *P* < 0.05; **indicates *P* < 0.01; ***indicates *P* < 0.001

ALND: Axillary lymph node dissection; BCT: Breast-conserving treatment; DFI: Disease-free Interval; Her-2, human epidermal growth factor receptor-2;

HR: Hormonal receptor; LN: Lymph node; LRR: Locoregional recurrence; NACT: Neoadjuvant chemotherapy treatment; SLNB: Sentinel lymph node biopsy;

TNBC: Triple-negative breast cancer.

**Supplementary table S3. Clinicopathological characteristics of low-risk and high-risk patients in the training cohort.**

| Variable | Low-risk group (%) *N*=266 | High-risk group (%) *N*=80 | *P* value |
| --- | --- | --- | --- |
| Age at the diagnosis of breast cancer, year | | | 0.010^*^ |
| ≤35 | 24 (9.0%) | 11 (13.8%) |  |
| 35-70 | 222 (83.5%) | 55 (68.8%) |  |
| ≥70 | 20 (7.5%) | 14 (17.5%) |  |
| Menopausal status |  |  | 0.463 |
| Premenopausal | 112 (42.1%) | 30 (37.5%) |  |
| Postmenopausal | 154 (57.9%) | 50 (62.5%) |  |
| Received NACT before surgery |  |  | <0.001^***^ |
| No | 229 (86.1%) | 43 (53.8%) |  |
| Yes | 37 (13.9%) | 37 (46.3%) |  |
| Initial breast operation |  |  | <0.001^***^ |
| BCT | 84 (31.6%) | 5 (34.4%) |  |
| Mastectomy | 182 (68.4%) | 75 (65.6%) |  |
| Pathology |  |  | <0.001^***^ |
| IDC | 184 (69.2%) | 74 (92.5%) |  |
| ILC | 5 (1.9%) | 0 (0%) |  |
| Others | 77 (7.5%) | 6 (7.5%) |  |
| Tumor size, cm |  |  | <0.001^***^ |
| ≤2.0 | 147 (55.3%) | 23 (28.8%) |  |
| >2.0 | 119 (44.7%) | 57 (71.3%) |  |
| Positive LN |  |  | <0.001^***^ |
| 0 | 155 (58.3%) | 9 (11.3%) |  |
| 1-3 | 56 (21.1%) | 26 (32.5%) |  |
| 4-9 | 34 (12.8%) | 25 (31.3%) |  |
| ≥10 | 21 (7.9%) | 20 (25.0%) |  |
| ER status |  |  | <0.001^***^ |
| Negative | 107 (40.2%) | 64 (80.0%) |  |
| Positive | 159 (59.8%) | 16 (20.0%) |  |
| PR status |  |  | <0.001^***^ |
| Negative | 128 (48.1%) | 67 (83.8%) |  |
| Positive | 138 (51.9%) | 13 (16.2%) |  |
| HR status |  |  | <0.001^***^ |
| Negative | 102 (38.3%) | 64 (80.0%) |  |
| Positive | 164 (61.7%) | 16 (20.0%) |  |
| HER-2 status |  |  | 0.002^**^ |
| Negative | 174 (65.4%) | 67 (83.8%) |  |
| Positive | 92 (34.6%) | 13 (16.2%) |  |
| DFI to LRR | | | <0.001^***^ |
| ≤2 year | 144 (54.1%) | 11 (13.8%) |  |
| >2 year | 122 (45.9%) | 69 (86.3%) |  |
| LRR sites |  |  | <0.001^***^ |
| Chest wall | 77 (28.9%) | 52 (65.0%) |  |
| Breast | 60 (22.6%) | 4 (5.0%) |  |
| Nodal recurrence | 114 (42.9%) | 10 (12.5%) |  |
| Multiple sites | 16 (6.0%) | 14 (17.5%) |  |

*indicates *P* < 0.05; **indicates *P* < 0.01; ***indicates *P* < 0.001

ALND: Axillary lymph node dissection; BCT: Breast-conserving treatment; DCIS: Ductal carcinoma in situ;

DFI: Disease-free Interval; ER: Estrogen receptor; Her-2, human epidermal growth factor receptor-2;

HR: Hormonal receptor; IDC: Invasive ductal carcinoma; ILC: Invasive lobular carcinoma; LN: Lymph node;

LRR: Locoregional recurrence; NACT: Neoadjuvant chemotherapy treatment; SLNB: Sentinel lymph node biopsy;

TNBC: Triple-negative breast cancer.

**Supplementary Table S4. Multivariate cox regression analysis of local treatment and systemic treatment for the post-LRR patients in low-risk and high-risk groups in the training cohort.**

| Variable | Overall survival (OS) | | |  | Overall survival (OS) | | |
| --- | --- | --- | --- | --- | --- | --- | --- |
|  | Hazard Ratio | 95% Confidence Interval | *P* value |  | Hazard Ratio | 95% Confidence Interval | *P* value |
| **For low-risk patients** | | |  |  | **For high-risk patients** | |  |
| **Local treatment** | |  |  |  |  |  |  |
| Resectable surgery after LRR | | |  |  |  |  |  |
| No | 1.000 (reference) | — | — |  | 1.000 (reference) | — | — |
| Yes | 0.548 | 0.319-0.942 | 0.029^*^ |  | 0.785 | 0.363-1.700 | 0.539 |
| Radiotherapy after LRR | | |  |  |  |  |  |
| No | 1.000 (reference) | — | — |  | 1.000 (reference) | — | — |
| Yes | 0.791 | 0.461-1.356 | 0.394 |  | 0.599 | 0.331-1.087 | 0.092 |
| **Systemic treatment** |  |  |  |  |  |  |  |
| Chemotherapy after LRR | | |  |  |  |  |  |
| No | 1.000 (reference) | — | — |  | 1.000 (reference) | — | — |
| Yes | 0.867 | 0.491-1.531 | 0.623 |  | 0.386 | 0.185-0.808 | 0.011^*^ |
| Anti-Her-2 target therapy after LRR | | |  |  |  |  |  |
| No | 1.000 (reference) | — | — |  | 1.000 (reference) | — | — |
| Yes | 1.153 | 0.648-2.051 | 0.629 |  | 0.529 | 0.265-1.057 | 0.072 |
| Hormonal therapy after LRR |  |  |  |  |  |  |  |
| Nodal recurrence | 1.000 (reference) | — | — |  | 1.000 (reference) | — | — |
| Multiple sites | 1.048 | 0.620-1.771 | 0.861 |  | 0.200 | 0.079-0.504 | 0.001^**^ |

*indicates *P* < 0.05; **indicates *P* < 0.01; ***indicates *P* < 0.001

Her-2, human epidermal growth factor receptor-2; LRR: Locoregional recurrence.

**Supplementary table S5. Univariate cox regression analysis of the Association Between Clinicopathologic Factors, and Overall Survival (OS) in different types of isolated local recurrence (breast or chest wall recurrence) in the training cohort.**

| Variable | Isolated breast recurrence (N=64) | | |  | Isolated chest wall recurrence (N=129) | | |
| --- | --- | --- | --- | --- | --- | --- | --- |
|  | Hazard Ratio | 95% Confidence Interval | *P* value |  | Hazard Ratio | 95% Confidence Interval | *P* value |
| Age of diagnosis of breast cancer, yrs | | |  |  |  |  |  |
| 35-70 | 1.000 (reference) | — | — |  | 1.000 (reference) | — | — |
| ≤35 | 2.967 | 0.888-9.908 | 0.077 |  | 1.414 | 0.633-3.152 | 0.399 |
| ≥70 | 0.000 | 0.000 | 0.988 |  | 2.685 | 1.285-5.612 | 0.009** |
| Menopausal status | | |  |  |  |  |  |
| Premenopausal | 1.000 (reference) | — | — |  | 1.000 (reference) | — | — |
| Postmenopausal | 2.681 | 0.799-8.993 | 0.110 |  | 1.508 | 0.873-2.605 | 0.141 |
| Receive NACT |  |  |  |  |  |  |  |
| No | 1.000 (reference) | — | — |  | 1.000 (reference) | — | — |
| Yes | 1.094 | 0.236-5.068 | 0.909 |  | 1.827 | 1.054-3.167 | 0.032* |
| Pathology | | |  |  |  |  |  |
| IDC | 1.000 (reference) | — | — |  | 1.000 (reference) | — | — |
| ILC | 0.738 | 0.234-2.332 | 0.605 |  | 2.185 | 1.073-4.449 | 0.031* |
| Others | 0.000 | 0.000 | 0.988 |  | 1.263 | 0.160-9.993 | 0.825 |
| Tumor size |  |  |  |  |  |  |  |
| ≤2.0 | 1.000 (reference) | — | — |  | 1.000 (reference) | — | — |
| >2.0 | 3.289 | 1.020-10.608 | 0.046* |  | 1.411 | 0.818-2.432 | 0.215 |
| Number of positive LNs | | |  |  |  |  |  |
| 0 | 1.000 (reference) | — | — |  | 1.000 (reference) | — | — |
| 1-3 | 1.636 | 0.435-6.151 | 0.467 |  | 2.386 | 1.145-4.974 | 0.020* |
| 4-9 | 6.527 | 1.404-30.343 | 0.017* |  | 2.077 | 1.034-4.170 | 0.040* |
| ≥10 | 0.000 | 0.000 | 0.989 |  | 2.372 | 1.171-4.805 | 0.017* |
| ER status | | |  |  |  |  |  |
| Negative | 1.000 (reference) | — | — |  | 1.000 (reference) | — | — |
| Positive | 1.018 | 0.321-3.227 | 0.976 |  | 0.361 | 0.210-0.620 | <0.001*** |
| PR status | | |  |  |  |  |  |
| Negative | 1.000 (reference) | — | — |  | 1.000 (reference) | — | — |
| Positive | 0.764 | 0242-2.417 | 0.647 |  | 0.312 | 0.169-0.574 | <0.001*** |
| HR status | | |  |  |  |  |  |
| Negative | 1.000 (reference) | — | — |  | 1.000 (reference) | — | — |
| Positive | 0.946 | 0.299-2.993 | 0.924 |  | 0.329 | 0.191-0.567 | <0.001*** |
| Her-2 status | | |  |  |  |  |  |
| Negative | 1.000 (reference) | — | — |  | 1.000 (reference) | — | — |
| Positive | 1.618 | 0.438-5.979 | 0.471 |  | 0.831 | 0.576-1.198 | 0.321 |
| Adjuvant radiotherapy | | |  |  |  |  |  |
| No | 1.000 (reference) | — | — |  | 1.000 (reference) | — | — |
| Yes | 0.869 | 0.274-2.753 | 0.811 |  | 0.894 | 0.535-1.494 | 0.670 |
| Adjuvant chemotherapy | | |  |  |  |  |  |
| No | 1.000 (reference) | — | — |  | 1.000 (reference) | — | — |
| Yes | 1.637 | 0.442-6.062 | 0.460 |  | 0.791 | 0.409-1.529 | 0.486 |
| Hormonal therapy | | |  |  |  |  |  |
| No | 1.000 (reference) | — | — |  | 1.000 (reference) | — | — |
| Yes | 1.239 | 0.390-3.943 | 0.716 |  | 0.447 | 0.262-0.764 | 0.003** |
| Anti-Her-2 target therapy | | |  |  |  |  |  |
| No | 1.000 (reference) | — | — |  | 1.000 (reference) | — | — |
| Yes | 0.502 | 0.065-3.899 | 0.510 |  | 0.584 | 0.303-1.127 | 0.109 |
| DFI to LRR | | |  |  |  |  |  |
| ≤2, yrs | 1.000 (reference) | — | — |  | 1.000 (reference) | — | — |
| >2, yrs | 0.614 | 0.165-2.279 | 0.466 |  | 0.358 | 0.215-0.595 | <0.001*** |

*indicates *P* < 0.05; **indicates *P* < 0.01; ***indicates *P* < 0.001

BCT: Breast-conserving treatment; DFI: Disease-free Interval; LRR: Locoregional recurrence.
